# T-Cell Redirecting Therapies in Multiple Myeloma: Pathogenesis and Management of Toxicities Beyond CRS and ICANS

**DOI:** 10.3390/cancers17091514

**Published:** 2025-04-30

**Authors:** Katia Mancuso, Marco Talarico, Enrica Manzato, Simona Barbato, Paola Tacchetti, Elena Zamagni, Michele Cavo

**Affiliations:** 1 IRCCS Azienda Ospedaliero-Universitaria di Bologna, Istituto di Ematologia “Seràgnoli”, 40138 Bologna, Italy; katia.mancuso3@unibo.it (K.M.); marco.talarico@studio.unibo.it (M.T.); enrica.manzato@studio.unibo.it (E.M.); simona.barbato3@unibo.it (S.B.); paola.tacchetti2@unibo.it (P.T.); 2 Dipartimento di Scienze Mediche e Chirurgiche, Università di Bologna, 40138 Bologna, Italy; michele.cavo@unibo.it

**Keywords:** multiple myeloma, immunotherapy, T-cell redirecting therapies, hematologic toxicities, cytopenias, immune effector cell associated hematotoxicity, hypogammaglobinemia, infections, immune effector cell-like lymphohistiocytosis syndrome

## Abstract

The introduction of chimeric antigen receptor T (CAR-T) cell and bispecific antibody (BsAb) therapies has revolutionized multiple myeloma (MM) treatment, offering exceptional efficacy, and culminating in recent regulatory approval. However, these therapies have brought unique toxicity challenges, manifesting not only with the well-established cytokine release syndrome (CRS) and immune effector cell-associated neurotoxicity syndrome (ICANS), but also with the emergence of other common and equally distinctive toxicities, including cytopenias, hypogammaglobulinemia, infections, and the rare but life-threatening immune effector cell-like lymphohistiocytosis syndrome (IEC-HS). These adverse events are characterized by unique mechanisms of action that differ from those of previous treatments for MM, thereby requiring specialized knowledge to optimize day-to-day management and ultimately maximize therapeutic benefits while ensuring patient safety. Additionally, the toxicity profiles of these T-cell engager therapies are becoming increasingly important in treatment decisions, with implications for patient selection and therapy sequencing. In this review, we provide a comprehensive overview of the current state-of-the-art regarding the incidence, etiopathogenetic mechanisms, and clinical manifestations of these increasingly less non-prototypical but still lesser-known side effects than CRS and ICANS, in order to offer clear and actionable insights into their effective management, while emphasizing critical points for future improvement, in view of the increasing number of MM patients who will benefit from the newly approved and upcoming immunotherapies.

## Simple Summary

Chimeric antigen receptor (CAR) T-cell therapy and bispecific antibody treatments have markedly advanced the clinical management of multiple myeloma, leading to improved patient outcomes. However, these innovative therapies are also associated with a spectrum of novel and often complex toxicities. In addition to the well-characterized cytokine release syndrome and immune effector cell-associated neurotoxicity syndrome, clinicians are increasingly encountering complications such as prolonged cytopenias, hypogammaglobulinemia, infections, and the rare but potentially life-threatening immune effector cell-associated hemophagocytic lymphohistiocytosis-like syndrome. These adverse effects are underpinned by distinct pathophysiological mechanisms and necessitate tailored management approaches. This review consolidates current evidence on these emerging toxicities, with the aim of informing clinical practice and supporting decision-making as the application of T-cell redirecting therapies continues to expand in the treatment landscape of multiple myeloma.

## 1. Introduction

The introduction of innovative therapies based on the use of chimeric antigens receptor T-cells (CAR-Ts) and bispecific antibodies (BsAbs) has completely revolutionized the therapeutic scenario of patients with multiple myeloma (MM). In particular, given the results achieved with these breakthrough therapies, two CAR-T products directed against the B-cell maturation antigen (BCMA), named idecabtagene vicleucel (ide-cel, Abecma^®^) and ciltacabtagene autoleucel (cilta-cel, CARVYKTI^®^), and three BsAbs directed either against BCMA (teclistamab, elranatamab) or the G protein-coupled receptor class C group 5 member D (GPRC5D) (talquetamab) have been rapidly approved in recent years [1,2,3,4,5,6].

Nevertheless, though the efficacy of these therapies in heavily pretreated MM is extremely encouraging, the emergence of unique and potentially life-threatening new toxicities, both in the context of clinical trials and in real-life, dampens some of the enthusiasm and alerts us to the need to learn how to recognize, manage, and possibly prevent these side effects. Among these, the cytokine release syndrome (CRS) and the immune effector cell-associated neurotoxicity syndrome (ICANS) have initially been the most feared adverse events (AEs), as the scientific community was mostly unprepared to handle such complications. As of today, however, very detailed guidelines and consensuses are currently available for the management of these now-known toxicities, and the wider use of preventive or early interventions, has intensely decreased the incidence of severe—or lethal -CRS and ICANS across all hematologic diseases over time [7,8,9]. In addition, intensive research is underway to reduce their incidence and severity further, including through the use of novel constructs with low CD3 affinity to reduce potential toxicities as a mitigation strategy [10,11].

By contrast, the widespread use of immunotherapies came with other relevant and frequent toxicities, with specific manifestations related to these products, such as cytopenias, infections, hypogammaglobulinemia, and the less frequent immune effector cell-like lymphohistiocytosis syndrome (IEC-HS). Initially less feared, these toxicities can actually give rise to severe complications that can compromise treatment efficacy and patients’ quality of life, as well as preclude further treatment, to the point of being fatal, presently accounting for the majority of non-relapse mortality. This review is therefore aimed at providing a comprehensive overview of the current state-of-the-art in terms of incidence, mechanisms, and clinical manifestations of these “less and less non-prototypical” side effects, to help provide clarity and useful details for their management, and ultimately maximize the benefits from the use of cutting-edge therapies that have already entered daily clinical practice in MM.

## 2. Cytopenias

Prototypical toxicities such as CRS and ICANS have been associated with CAR-T-cell and BsAbs therapies since their advent in the clinics. Nevertheless, hematologic toxicities represent the most common AEs encountered with the use of these products, both in the context of clinical trials and in real-life, regardless of the agent in use and the target antigen [12,13].

In particular, the acronym ICAHT (immune effector cell associated hematotoxicity) has recently been proposed to define cytopenias occurring after CAR-T-cell therapy, which differs from chemotherapy-related cytopenias. More in detail, a joint consensus by the European Society of Hematology (EHA) and the European Society for Blood and Marrow Transplantation (EBMT) has differentiated an early ICAHT (occurring within the first 30 days after CAR-T-cell infusion) from a late ICAHT (occurring beyond day +30 after infusion). Due to the major role of neutropenia in this setting, with isolated anaemia or thrombocytopenia representing rare events, a classification system to grade ICAHT was developed, based on its depth and, for early ICAHT only, duration [12] (Table 1).

Overall, ICAHT showed a remarkable clinical impact, with hypogammaglobulinemia and lymphocyte exhaustion, on infectious risk. Moreover, ICAHT increases patient morbidity and waste of healthcare resources, through haemorrhagic diathesis, transfusion dependency, and need for hospitalisation. Indeed, severe ICAHT has been related to a significant increase in the incidence of severe bacterial infections, with a high rate of life-threatening and fatal infectious events, thereby increasing the non-relapse mortality (NRM) rate [9,14,15,16]. Notably, in this sense, results from a recent retrospective analysis showed that the correlation between neutropenia and infections appears to be more significant in patients undergoing CAR-T therapies than in those treated with BsAbs, though in the absence of neutropenia, the infection rate was higher with BsAbs [17].

### 2.1. Incidence of Cytopenias from Clinical Trials and Real-World Setting

As anticipated, cytopenias are the higher-grade (i.e., grade ≥ 3) toxicities most reported across all pivotal clinical trials with both CAR-Ts and BsAbs-based therapies (for details, see Table 2) and in real-world evidence.

More in detail, grade ≥ 3 neutropenia occurred in 89% and 76% of patients treated with ide-cel in the KarMMa and KarMMa-3 trials, respectively, lasting more than one month in about 40% of cases (median time to recovery [TTR] to grade ≤ 2 was 1.9 and 1.7 months, respectively). In addition, grade ≥ 3 thrombocytopenia was experienced by 52% (48% persistent over 1 month; median TTR to grade ≤ 2 of 2.1 months) and 42% (37% persistent over 1 month; median TTR of 1.9 months) of patients in the two trials, respectively, while severe anaemia was reported in 60% and 51% of patients in the same trials [1,2,18]. As for the use of ide-cel outside clinical trials, experiences from real-life confirmed a high incidence of ICAHT, with grade ≥ 3 neutropenia occurring in up to 100% of patients, grade ≥ 3 thrombocytopenia in up to 75% and grade ≥ 3 anaemia in up to 94% (no significant difference observed based on patient age), and persistence beyond 1 month after infusion in up to 60%, 59% and 38% of patients, respectively. Additionally, some retrospective studies reported persistence of grade ≥ 3 cytopenias beyond 3 and 6 months after infusion (in up to 25% and 10% for neutropenia, 36% and 24% for thrombocytopenia, and 15% and 10% for anaemia, respectively) [19,20,21,22,23,24].

**Table 2:** Incidence of cytopenias, hypogammaglobulinemia and infections in pivotal trials.

	Ide-Cel		Cilta-Cel		Teclistamab	Elranatamab	Talquetamab	
	KarMMa [1]	KarMMa-3 [2,18]	CARTITUDE-1 [25,26,27]	CARTITUDE-4 [3,28]	MajesTEC-1 [4,29,30]	MagnetisMM-3 [5]	MonumenTAL-1 [6,31,32]	
							405 μg QW	800 μg Q2W
**Neutropenia**								
All grade	91%	78%	96%	90%	72%	49%	67%	36%
Grade ≥ 3	89%	76%	95%	90%	65%	49%	60%	32%
Persistent * grade ≥ 3	41%	40%	30%	26%	/	/	/	/
**Thrombocytopenia**								
All grade	63%	54%	79%	54%	42%	31%	37%	23%
Grade ≥ 3	52%	42%	60%	41%	23%	24%	23%	11%
Persistent * grade ≥ 3	48%	37%	41%	26%	/	/	/	/
**Anaemia**								
All grade	70%	66%	81%	54%	55%	49%	60%	42%
Grade ≥ 3	60%	51%	68%	36%	38%	37%	30%	23%
**Hypogammaglobulinemia**								
All grade ^§^	21%	11%	12%	42%	74.5%	75.5%	87%	71%
**Infections**								
All grade	69%	58%	58%	62%	80%	70%	58%	65%
Grade ≥ 3	22%	24%	20%	27%	55%	40%	22%	16%
Grade 5	UR	4%	4%	6%	13%	6.5%	<1.5%	
**IEC-HS**								
	3%		1%		No cases reported			

* Persistent: >1 month. ^§^ Definition of hypogammaglobulinemia varies across different trials. Abbreviations: QW = every week; Q2W = every other week; UR = unreported; IEC-HS = immune-effector cell-associated hemophagocytic lymphohistiocytosis-like syndrome.

Similarly, the use of cilta-cel within clinical trials resulted in grade ≥ 3 neutropenia in 95% (CARTITUDE-1) and 90% (CARTITUDE-4) of patients, with >1 month persistence in 30% and 26%, respectively. In addition, grade ≥ 3 thrombocytopenia was reported in 60% and 41% of patients (>1 month in 41% and 26%, respectively), and grade ≥ 3 anaemia in 68% and 36% of patients. Persistence of cytopenias after 2 or 3 months from infusion was also reported (neutropenia 10%; thrombocytopenia 26%) [3,25,26]. Experiences from the real-world also confirmed the high rate of prolonged hematologic toxicity, with 70–75% of patients experiencing grade ≥ 3 cytopenias 1 month after CAR-T. In particular, grade ≥ 3 neutropenia was observed in 59%, 20% and 11% of patients at 1, 2 and 3 months after infusion, and grade ≥ 3 thrombocytopenia in 49%, 22% and 14% of patients, respectively [33,34].

In addition to these findings, recent data from pivotal phase I and II trials using GPRC5D-directed CAR-T therapies, though not yet approved, have shown similar rates of cytopenias, with grade ≥ 3 neutropenia, thrombocytopenia and anaemia occurring in up to 100%, 90% and 70% of patients, respectively [35,36,37,38]. Relative to these, a median duration of 14 days was reported for neutropenia, 24 days for anaemia, and 29 days for thrombocytopenia [38].

Noteworthy, the incidence and severity of cytopenias have also been reported to be high with T-cell-engaging BsAbs, although they appear to happen less frequently than with CAR-Ts (Table 2). Indeed, pivotal studies of the two anti-BCMA BsAbs, teclistamab and elranatamab, revealed a grade ≥ 3 neutropenia of 65% and 49%, thrombocytopenia of 23% and 24%, and anaemia of 38% and 37%, respectively [4,5,29,30]. The median time to onset of grade ≥ 3 neutropenia or febrile neutropenia with teclistamab was 2.3 months and the median duration of neutropenia was 1.2 months (0.8 months for grade ≥ 3 event) [29]. Similar to data reported in the context of clinical trials, the use of teclistamab in clinical practice showed an incidence of neutropenia up to 55% (grade ≥ 3 up to 38%), thrombocytopenia up to 63% (grade ≥ 3 up to 26%), and anaemia up to 80.5% (grade ≥ 3 up to 32%) [39,40].

Notably, hematologic toxic effects were the most common grade 3 or 4 AEs even in patients treated with the BsAb against the novel target GPRC5D. Among patients receiving talquetamab at the two-dosing schedules of 405 μg/kg weekly (QW) or 800 μg/kg every other week (Q2W) in the MonumenTAL-1 trial, grade ≥ 3 neutropenia was noted in 60% and 32% of patients, grade ≥ 3 thrombocytopenia in 23% and 11% of patients, and grade ≥ 3 anaemia in 30% and 23% at each dose, respectively. However, cytopenias were reversible and mostly limited to patients who had received step-up doses and early full doses through cycle 2 [6]. Because of their more recent approval, real-life experiences with the use of elranatamab and talquetamab in clinical practice yet are limited.

Overall, despite the absence of a head-to-head comparison, the above data suggest that cytopenias are more prevalent with BCMA-targeting BsAbs than with non-BCMA-directed agents and seem to be more likely to occur early in treatment.

### 2.2. Pathophysiology and Factors Contributing to Cytopenias

The pathophysiology of ICAHT remains incompletely understood, but some potential mechanisms have been proposed. In this sense, the timing of the occurrence of cytopenias in relation to CAR-T-cell infusion may contribute to defining the possible aetiology and related treatment. Patients usually experience a nadir in count within the first week after infusion, followed by a rapid complete hematologic recovery, likely due to non-myeloablative lymphodepletion chemotherapy. However, most patients experience a subsequent nadir (typically some weeks after infusion, with a biphasic or intermittent course characterized by recovery followed by a second or multiple decline(s) in absolute neutrophil counts, ANC). On the other hand, a minority of patients experience an aplastic course, with incomplete hematologic recovery and suboptimal response to growth factors, although aplastic phenotype and severe ICAHT are reported more frequently in lymphomas [41]. Indeed, though a comparison of clinical trials evaluating CAR-Ts is difficult, mainly due to the substantial heterogeneity of diseases, CAR-T products (CD28ξ vs. 4-1BBξ), and reporting of cytopenia, data from a recent retrospective study showed an incidence of severe ICAHT of 28% in mantle cell lymphoma, 23% in large B-cell lymphoma (LBCL) and 15% in MM [14].

Based on the variety of clinical presentations, cytopenias arising after the new immunotherapies may be caused by multiple mechanisms, that may also cut across various hematologic diseases and posited mostly by studies of lymphomas, a setting for which the use of CAR-Ts originates further back in time, and with more widespread use. More in detail, these factors span from malignancy-related characteristics, baseline bone marrow features, factors intrinsic to CAR-T and related toxicities, or baseline inflammatory status [42,43,44,45,46].

Among these, major factors contributing to a poor haematopoietic reserve include previous therapies, albeit there are conflicting data regarding the number of prior lines of therapy or prior stem cell transplant (SCT), and increased marrow disease burden, likely due to local inflammatory stress related to CAR-T-cell activation [43]. In addition, a possible role of pre-existing clonal haematopoiesis of indeterminate potential (CHIP) remains to be clarified. Indeed, an expansion of CHIP clones has been observed following CD19-directed CAR-T therapy, with a trend towards more pronounced late cytopenias, though data in relapsed/refractory MM (RRMM) patients are still limited [47,48]. In this regard, a recent single-centre retrospective study on 104 patients receiving anti-BCMA CAR-T therapy (with 55% of patients having a diagnosis of CHIP prior to infusion) found no correlation with the incidence or severity of cytopenias or with the onset of therapy-related myeloid neoplasms (t-MN), but a significant risk for prolonged cytopenias was observed [49].

Moreover, it has been proposed that infections or other causes of inflammatory state (including high-grade CRS) may also play a role [50]. A recent experience with BCMA-directed CAR-T-cell therapy observed a paracrine effect on inhibiting the differentiation of hematopoietic stem and progenitor cells and promoting more immature phenotypes [46]. Furthermore, the use of tocilizumab has been reported as a risk factor for prolonged neutropenia, likely due to the inhibition of migration of neutrophils from the marginal pool to the bloodstream. Conversely, other neutrophil functions, including phagocytosis, respiratory burst and chemotaxis, do not seem altered by the anti-IL6R drug [51,52]. Other factors associated with ICAHT include longer CAR-T-cell persistence (without apparent correlation to expansion peak), malignancy relapse, onset of secondary t-MN and development of rare complications including IEC-HS or thrombotic microangiopathy (particularly in patients with previous SCT) [53,54,55,56].

The CAR-HEMATOTOX (HT) score, initially proposed for use across LBCL patients to predict the risk of developing severe haematotoxicity and complications after therapy, was retrospectively validated in 113 RRMM patients receiving anti-BCMA CAR-Ts. This score is calculated prior to lymphodepletion and incorporates both factors related to baseline hematopoietic reserve (e.g., haemoglobin, platelet count and ANC) and baseline inflammatory state (i.e., C-reactive protein and ferritin) [41]. Compared to low-risk patients (score 0–1), high-risk patients (score ≥ 2) experienced prolonged severe neutropenia (median 9 vs. 3 days), increased rates of severe infection (40% vs. 5%), higher non-relapse mortality (13% vs. 2%, primarily imputable to fatal infections) and inferior treatment outcomes in terms of median PFS (5 vs. 15 months) and median OS (10.5 months vs. NR) [57]. A recent study on RRMM patients receiving BCMA-directed CAR-T-cell therapy confirmed the prominent role of high peak inflammatory markers and baseline cytopenias, which resulted in being the best predictive biomarker for prolonged haematological toxicity [46]. However, future prospective validation remains necessary to enhance the predictive power of this model as a potential risk-stratifier for severe toxicity and clinical outcomes.

Among patients treated with BsAbs, the kinetics of cytopenias is yet to be established, even though they appear more common earlier during treatment, while the underlying aetiology and pathophysiologic mechanisms are even more enigmatic. Considering the heavy pre-treatment status of patients currently treated with these agents, a poor hematopoietic reserve was proposed as a major contributor, though the temporal effect of cytopenias following administration of BsAbs and hematologic recovery following dose delays suggests a relation to treatment itself, likely due to a “bystander” effect from therapy-induced cytokines release and pro-inflammatory state [9,58,59]. A recent pooled analysis did not observe significant differences between BsAbs targeting BCMA or other antigens in terms of all grades and types of hematologic toxicities, but grade ≥ 3 cytopenias resulted in higher in patients receiving anti-BCMA BsAbs [60].

### 2.3. Diagnostic Work-Up and Management of Cytopenias

The diagnostic approach to patients experiencing hematological toxicity after CAR-T-cell infusion or during treatment with BsAbs requires an incremental work-up. For patients with severe cytopenia before lymphodepletion, a histopathological bone marrow evaluation and assessment of disease infiltration can assist in the interpretation of subsequent cytopenia trajectories and lead the therapeutic management [43]. Thus, blood count should be closely monitored for the first 6–12 months after CAR-Ts and throughout the entire duration of treatment with BsAbs [8,9].

In patients with persisting severe neutropenia after CAR-T-cell infusion (e.g., beyond day 10), it is necessary to check for concomitant myelotoxic medications, to rule out active infections or vitamin deficiency and to exclude IEC-HS by dosing serum ferritin and triglycerides. In case of grade ≥ 3 cytopenias beyond day +14 or refractoriness to granulocyte colony-stimulating factor (G-CSF), advanced viral studies and bone marrow aspiration and biopsy should be considered. In case of persistent aplasia beyond 1 month or new-onset cytopenias or refractoriness to therapeutic approaches, it is recommended to exclude malignancy progression or t-MN by bone marrow examinations including immunohistochemistry/flow cytometry, cytogenetics, and next-generation sequencing [12,43].

Regarding the therapeutic approach to immunotherapies-related cytopenias in RRMM, some guidelines and recommendations (summarized in Table 3) have recently been proposed [7,8,9]. In particular, the management of ICAHT can be distinguished into an initial phase (addressing early expected cytopenias) and a later phase (in case of persistent or therapy-refractory cytopenias). Notably, the introduction of the HT score may help identify patients at higher risk for prolonged high-grade neutropenia and severe infections, allowing for risk-adapted management. In addition, due to the frequent presence of severe anaemia and thrombocytopenia, transfusions of packed red blood cell (RBC) concentrates, and platelets are an essential part of supportive care. In this sense, the EHA/EBMT consensus recommends irradiation of blood products from 7 days before leukapheresis until at least 90 days after CAR-T-cell infusion to prevent transfusion-associated graft-versus-host disease [12].

Regarding neutropenia, supportive therapy with G-CSF is the mainstay of treatment, although the timing of treatment initiation and its use during toxicities are still debated. Results from preclinical models seem to associate the use of growth factors to an increased inflammatory state, with a potential risk of promoting CRS and ICANS, while data from clinical trials are lacking, as the use in the first two weeks is not allowed in most studies [61,62]. However, in retrospective real-life studies—mainly focused on lymphomas—early use of G-CSF has shown an acceptable safety profile, having been correlated with faster neutrophil recovery, reduced risk of febrile neutropenia and comparable CAR-T expansion and treatment outcomes. Of note, G-CSF administration correlates with an increased risk of grade ≥ 2 CRS, though its use in patients with grade 1 CRS does not appear to worsen the condition [63,64,65,66,67].

Unlike previously reported, a single retrospective experience showed worse treatment responses and survival in patients receiving G-CSF after CAR-T-cell therapy [68]. Its use is recommended in case of severe neutropenia (ANC < 500/μL), with or without infectious complications, at 5 μg/kg once daily, but higher doses may be considered in the absence of response. In patients at higher risk for severe ICAHT (high HT score or presence of other risk factors), prophylactic use of G-CSF from day +2 may be considered. As reported, the majority of patients (>80%) respond to G-CSF, but the ones with biphasic course of neutropenia may require intermittent support [42,69]. Conversely, patients developing an aplastic course of cytopenias are usually refractory to G-CSF (absence of count recovery despite ≥5 days of support or beyond day +14 from CAR-T-cell infusion) and require more advanced diagnostic work-up, as previously described [12,41,42]. In these cases, if a cryopreserved graft from prior treatments is available, a haematopoietic cell boost represents an encouraging strategy due to favourable safety profile and promising engraftment rates, with significant improvement of cytopenias and no adverse effects on patient outcome [70,71,72,73,74,75]. Another possible approach, particularly in cases with associated thrombocytopenia, is represented by thrombopoietin receptor agonists (TPO-RA): improvement of platelet count has been observed (with some studies also reporting an increase of ANC and haemoglobin), without significant toxicities [76,77,78,79]. Conversely, if an inflammatory trigger (e.g., severe CRS, IEC-HS) is considered causative of ICAHT, anti-inflammatory strategies (pulse-dose corticosteroids and/or anti-cytokine therapies such as tocilizumab or anakinra) are recommended [12,42]. Finally, in cases without adequate response to the above strategies, allogeneic haematopoietic stem cell transplantation (allo-HCT) represents the last resort, although anecdotal experiences are reported only in lymphomas and acute lymphoblastic leukaemia. In particular, if grade 4 ICAHT persists beyond day +30, a search for a donor is recommended. In this regard, a reasonable time frame for evaluating this approach could be 3 to 6 months after CAR-T infusion, to balance the possibility of a spontaneous gradual count recovery—which can occur over months after infusion—the risk of severe infections, the eradication of CAR-T-cells by graft and potential tumour control by the conditioning regimen and the graft-versus-tumour effect [12,42].

Management of cytopenias in patients receiving BsAbs includes transfusions of packed RBC or platelet concentrates if clinically required, G-CSF support (which should be administered in patients experiencing grade ≥ 3 neutropenia but should be avoided when patients are at major risk of CRS), and eventual TPO-RA or erythropoiesis stimulating agents as per institutional guidelines. In the case of grade 4 neutropenia or febrile neutropenia, however, administration of BsAbs should be withheld until resolution [7,9,80].

## 3. Hypogammaglobulinemia

Hypogammaglobulinemia represents an important on-target off-tumor toxicity of CAR-T-cell therapies and BsAbs in MM, because of BCMA and GPRC5D expression on almost all normal plasma cells. Moreover, BCMA is crucial for the survival of long-lived plasma cells, which further elucidates the deep and durable plasma cell aplasia described with BCMA-targeting therapies [80,81,82]. Additionally, hypogammaglobulinemia in patients affected by RRMM is a consequence of the malignancy itself and of previous treatments, especially anti-CD38 antibodies [83,84,85].

A retrospective study in patients receiving BCMA-targeting CAR-T-cell therapy observed an aplasia of bone marrow normal plasma cells in all patients, with a median duration of 7 months following infusion. An even longer period of hypogammaglobulinemia (especially for IgA) was reported (nadir at day 60 and median time to IgG, IgM and IgA recovery of 386 days, 254 days and not reached at a median follow-up of 16 months, respectively) [86].

Hypogammaglobulinemia significantly contributes to increased infectious risk: depletion of IgG2 and IgG1/IgG3 subclasses have been associated with a higher risk of bacterial and viral infections, respectively [87,88,89]. Among BCMA-directed CAR-T cell therapy recipients, patients with severe infections have lower serum IgG concentration than patients experiencing mild or moderate infections, and infectious events (especially respiratory ones) usually occur during periods of hypogammaglobulinemia [86,90,91]. Retrospective experiences in patients receiving BCMA and GPRC5D-directed BsAbs confirmed a significant association between infectious risk and severity of hypogammaglobulinemia, worsening during treatment [92,93].

### 3.1. Incidence of Hypogammaglobulinemia from Clinical Trials and Real-World Setting

Hypogammaglobulinemia is a common toxicity of CAR-T-cell therapies and BsAbs, as emerged by clinical trials (Table 2) and confirmed in the real-world setting. As its definition varies across different studies, it may be difficult to compare data. Overall, reported rates of hypogammaglobulinemia in the KarMMa and KarMMa-3 studies were 21% (with immunoglobulin replacement therapy, IgRT, in 62% of patients) and 11% (6.7% < 3 months after infusion, 1.4% at 3–6 months after infusion, 2.5% > 6 months after infusion), respectively [1,18]. Similar rates were confirmed among patients receiving ide-cel in real-life, with a rise of hypogammaglobulinemic patients from baseline (55%) to post-infusion (74%), a significant decrease in IgG levels and an increase of infections; in addition, the need for IgRT in up to 32% of patients was reported [22,23,94].

As for cilta-cel, laboratory findings of IgG serum concentration < 500 mg/dL were observed in 92% and 91% of patients in the CARTITUDE-1 and CARTITUDE-4, respectively, though hypogammaglobulinemia was reported as an adverse event in 12% and 42% of patients, with 66% of patients requiring IgRT in the latter trial [3,27].

Similarly, hypogammaglobulinemia was reported in most cases with the use of BsAbs within trials and in 100% of patients receiving anti-BCMA BsAbs in real-life [93]. Indeed, IgG serum concentration < 500 mg/dL was reported in 74.5% of patients receiving teclistamab in the MajesTEC-1 (46% of patients receiving IgRT), 87% and 71% of patients on treatment with talquetamab (QW 405 μg or Q2W 800 μg schedule, respectively) in the MonumenTAL-1, and <400 mg/dL in 75.5% of patients treated with elranatamab in the MagnetisMM-3 trial (43% of whom received IgRT) [4,5,6,29] On the other hand, data on the incidence of hypogammaglobulinemia after GPRC5D-directed CAR-T-cell therapy have not yet been reported.

### 3.2. Management of Hypogammaglobulinemia

Current guidelines and expert consensuses (Table 3) recommend monitoring of serum immunoglobulin concentration every 4 weeks and IgRT (400–500 mg/kg every 4 weeks) if the serum IgG concentration is <400 mg/dL (subtracting the amount of monoclonal protein, if IgG isotype), or in case of higher concentration but severe and/or recurrent infections, for both recipients of CAR-T-cell therapy and BsAbs.

Experts recommendations are homogeneous regarding the goal of maintaining IgG concentration > 400 mg/dL in both settings. However, different time-points for starting and pursuing IgRT are reported. For example, the International Myeloma Working Group (IMWG) suggests beginning the support before CAR-T-cell infusion and continuing for at least the first 3–6 months, whereas the Academic Consortium to Overcome Multiple Myeloma through Innovative Trials (COMMIT) recommends starting IgRT from 30 days after CAR-T infusion until 1 year [7,8,80]. Similarly, for patients receiving BsAbs, COMMIT recommends IgRT from the second month of treatment (preventing possible confusion between CRS and infusion reaction) whereas other guidelines do not specify a time-point. Conversely, there is agreement that IgRT should be pursued until the end of therapy or further, if serum IgG concentration is <400 mg/dL [7,8,9,80,95]. These recommendations are based on retrospective studies showing a lower risk of severe infections in patients treated with BsAbs receiving IgRT [93,96], though similar studies in the setting of CAR-T-cell therapies showed discordant results [90,97].

## 4. Infections

Infections represent an important clinical issue in patients receiving T-cell-engaging immunotherapies, as both clinical trials and real-world experiences have reported a high risk of severe infections and infection-related NRM.

A fundamental difference between novel immunotherapies is the duration of treatment, as CAR-Ts are a one-time therapy, while BsAbs are provided as a continuous treatment. As a result, the infectious risk is particularly high in the first 100 days after infusion in patients receiving CAR-T-cell therapies and constant throughout therapy in patients receiving BsAbs, while its duration after treatment discontinuation remains unclear [29,80,98].

### 4.1. Incidence of Infections from Clinical Trials and Real-World Setting

As reported by pivotal studies and confirmed by real-world data, during or after treatment with CAR-Ts and BsAbs, infections are frequent (for details, see Table 2).

Specifically, the infection rate of any grade ranges from 58 to 69% (grade ≥ 3 20–27%) in patients receiving anti-BCMA CAR-Ts and fatal infections were reported in 4–6% of patients [1,2,3,25,27]. These data were further confirmed by several real-world experiences, reporting an overall incidence of up to 58% of patients (grade ≥ 3 up to 23%) [20,23,34,86,90,99].

Notably, infection rates can vary widely, especially regarding the observation time, and this may cause potentially biased comparisons. For example, the continuous treatment with BsAbs is associated with a cumulative risk of infections, not showing a plateau [17]. Therefore, a harmonized reporting model of infections for patients receiving BsAbs, based on reporting all infections observed in an individual patient per observation time, has been recently proposed [100]. In addition to this, a potential confounding factor could be determined by the COVID-19 pandemic. Indeed, the use of teclistamab and elranatamab has been associated with 70–80% of infections (40–55% of grade ≥ 3) within trials [4,5,29]. Importantly, the enrolment period of the MajesTEC-1 trial was concurrent with the peak of the pandemic (March 2020–August 2021), resulting in 13% of fatal infectious in patients treated with teclistamab, mostly (11%) due to COVID-19 [4,29]. Conversely, subsequent data on elranatamab showed grade 5 infections in 6.5% of patients in MagnetisMM-3, which enrolled patients after the pandemic (February 2021–January 2022), with 1.6% of deaths caused by COVID-19 [5]. Moreover, although real-life experiences with teclistamab have confirmed a high rate of infectious events (up to 54.5% of patients; up to 27% grade ≥ 3), it was relatively lower than in the trial, likely due to the attenuated hazard of COVID-19 [39,40,93].

Of note, by changing the target of BsAbs, the rate of infections may vary considerably with respect to anti-BCMA agents. Indeed, in the MonumenTAL-1, infections occurred in 58% and 65% of patients receiving QW 405 μg and Q2W 800 μg talquetamab, respectively. However, grade ≥ 3 infections were reported in 22% and 16% of patients and less than 1.5% of patients died from infections [31,32]. Similarly, the risk of infection with Fc receptor homolog 5 (FcRH5)-targeting BsAb resulted lower (54% any grade; 19% grade ≥ 3), likely due to less severe neutropenia (31% all grades; 28% grade ≥ 3) and hypogammaglobulinemia [101]. In line with this, data from a recent real-world retrospective experience with teclistamab, elranatamab and talquetamab showed a significant difference in patients receiving BCMA-directed BsAbs (73%) as compared to those receiving talquetamab (51%) [102], as also confirmed in recent pooled- and meta-analyses including more than 1000 and 1600 patients, respectively [103,104]. This difference may likely be explained by the high expression of BCMA on normal plasma cells, where it plays an important role in survival and downstream signalling [81]. Furthermore, the risk for severe infections in patients receiving the GPRC5D-directed BsAb has been described especially during the first cycles of therapy whereas it is more constant with BCMA-directed BsAbs [32].

Interestingly, a retrospective comparison between BCMA-directed CAR-T-cell therapies and BsAbs showed an increased susceptibility to severe infections and a more persistent infectious risk with BsAbs than with CAR-T (40% vs. 26%), including fatal infections (7% versus 0%). In addition, patients receiving CAR-T-cell therapy showed a higher risk for severe infections in the first 100 days after infusion (79% versus 50%), with a reversion of this pattern after 6 months. During periods of hypogammaglobulinemia, patients receiving BsAbs showed a higher rate of any grade infections and severe infections than patients treated with CAR-Ts [17].

### 4.2. Pathophysiology and Risk Factors

Patients receiving T-cell-engaging immunotherapies are characterized by severe immunosuppression, partially due to the malignancy itself and prior treatments (particularly SCT and CD38-directed therapies), and partly emerging as major toxicity resulting from these new immunotherapies.

Several underlying mechanisms have been recognized: leukopenia (both neutropenia and lymphopenia) and hypogammaglobulinemia, which have been previously described, T-cell exhaustion and the unique inflammatory toxicities of these novel immunotherapies [90,104,105]. In particular, CRS and ICANS have been proposed as etiologic mechanisms of immunosuppression due to the massive cytokines release and consequent immune paralysis; their severity and related treatments (particularly corticosteroid administration) have been proposed as etiologic mechanisms of immunosuppression and related to the infectious risk, especially during the first 90 days following infusion, though a recent experience with ide-cel showed discordant results [90,100,102,106,107,108]. Furthermore, several evidence shows that chronic T-cell stimulation due to continuous treatment with BsAbs is causative of T-cell exhaustion, which is considered a mechanism of resistance to such therapies and plays a fundamental role in the pathogenesis of infections, with immunological improvement achieved through treatment-free intervals [109,110,111,112].

An attempt to stratify patients receiving CAR-T therapies according to the infectious risk was recently proposed by a retrospective analysis with CD19-targeting CAR-T-cells. Specifically, the integration of HT score and procalcitonin concentration on the first day of fever after infusion could identify patients at higher risk of severe infections during coincident CRS [113]. However, this model has not been validated in MM, and similar tools for infectious risk in this setting are lacking.

### 4.3. Aetiologies and Infection Sites

Unlike CD19-targeting CAR-T-cell therapies, after which bacterial infections are the most common, the aetiologies of infections following BCMA-directed CAR-Ts seem to vary upon patient follow-up.

Early infections occur within 30 days after infusion, accounting for 11–38% of infections in different experiences (with discordant data regarding the predominance of bacterial or viral aetiologies, described in 43–68% and 27–57% of cases, respectively), and severe infectious events are mostly reported in this time-frame [23,86,90,99]. Specifically, early bacterial infections usually occur during the first two weeks of neutropenia, manifesting as bacteraemia or organ-specific infections (lungs, respiratory tract, skin and soft tissues represent the most commonly affected sites) [23,86,90]. *C. difficile* colitis (due to hospitalization and antimicrobial administration) has also been described as a common early infection in this setting [23]. Late infections, usually less severe, represent up to 89% of infectious events following CAR-Ts and appear to be similarly distributed between day +30 to day +100 after infusion and after day +100, although patients with a more complicated course and hospitalization that extends beyond day +30 may have a different infectious risk than discharged patients [90,98].

In the first period, bacterial and viral infections primarily affecting the respiratory tract appear to be equally represented, whereas viral infections predominate thereafter, likely due to prolonged lymphopenia and hypogammaglobulinemia [90]. Fungal infections are quite rare, with most cases—predominantly yeast and invasive fungal infections—reported in the late setting and related to prolonged neutropenia, long-term corticosteroid use, and severe CRS and ICANS [23,90]. *Pneumocystis jirovecii* (PJP) infections have been rarely reported, likely because of effective prophylaxis [90]. The incidence and clinical relevance of cytomegalovirus (CMV) infection in this setting are unclear: most patients improve spontaneously, although rare cases of CMV end-organ disease have been described [114,115,116]. The risk of HBV reactivation in patients receiving adequate prophylaxis is considered low, but a few fatal cases have been reported [117,118].

Among patients receiving BsAbs, a progressively increasing risk of infections due to continuous treatment has been reported [92]. Different aetiologies have been described, with bacterial infections being the most commonly reported in a large retrospective experience, followed by viral, fungal and parasitic infections (56%, 38%, 5% and 1% respectively), though other studies reported a predominance (up to 58%) of viral infectious events [93,102,119]. Similar to patients receiving CAR-Ts, respiratory tract infections are prevalent, followed by systemic, genitourinary, gastrointestinal and skin/soft tissue infections [92,93,103]. Opportunistic infections (including CMV, PJP, aspergillosis, adenoviral pneumonia, oesophagal candidiasis and even progressive multifocal leukoencephalopathy) have also been described [93,102,103,104]. Regarding CMV, the development of viraemia has been reported in about 5% of patients receiving BsAbs, but the incidence of organ disease appears low [9,102].

### 4.4. Management of Infectious Risk

Several consensuses and guidelines to mitigate the infectious risk in patients receiving T-cell-engaging immunotherapies have been proposed (Table 4) [7,8,9,80,120].

Overall, baseline serologic screening for CMV, Epstein-Barr virus (EBV), hepatitis B and C viruses (HBV, HCV), and human immunodeficiency virus (HIV) to identify patients with active infection or at high risk for viral infection reactivation, and subsequent monitoring in positive cases are recommended [7,8,80]. The IMWG also recommends the evaluation of parvovirus B19 serology in endemic regions [9,80,120]. Moreover, high-risk patients (CMV seropositive, patients receiving prolonged and/or high doses of corticosteroids, siltuximab, anakinra or >1 dose of tocilizumab) experiencing unexplained fever or clinical conditions potentially involving CMV (pneumonia, colitis, hepatitis, cytopenias) should undergo blood polymerase chain reaction (PCR) testing to exclude its reactivation [80]. HBV carriers (positive HBsAg) or patients with a previous history of HBV infection should receive prophylaxis with Entecavir or Tenofovir and close monitoring of HBV titers and liver function [80,120]. In addition, proper management of neutropenia and hypogammaglobulinemia, as previously described, is essential in reducing the severe infectious risk associated with these novel immunotherapies.

Regarding patients treated with CAR-T, recent consensuses and guidelines recommend antiviral (valacyclovir 500 mg twice a day or acyclovir 400–800 mg twice a day) and PJP prophylaxis (co-trimoxazole 480 mg once daily or 960 mg 3 times each week) for all patients, from lymphodepletion until 6 months-1 year after infusion and/or CD4+ count > 200/μL [7,70,80,120]. Conversely, a risk-adapted strategy is proposed for antibacterial and antifungal prophylaxis, to be considered in patients at high risk for severe ICAHT (e.g., in the presence of a high HT score) once ANC is <500/μL and discontinued in the presence of a stable count recovery [7,12,70,80,120]. The European Myeloma Network (EMN) also advises the use of antibacterial prophylaxis for patients having a history of recurrent bacterial infections or hypogammaglobulinemia, according to institutional standards, based on local bacterial epidemiology (fluoroquinolones prophylaxis is the most commonly used) [7]. Additional risk factors to be considered for antifungal prophylaxis are represented by concomitant treatment with corticosteroids, prior allo-HCT, multiple immunosuppressive medication use (i.e., siltuximab and/or anakinra) and prior invasive aspergillosis [7,70,80,120]. Antifungal prophylaxis should include fluconazole or echinocandins, considering posaconazole or voriconazole in patients at higher risk for mould infections [7,80,120].

On the other hand, all patients treated with BsAbs should receive antiherpetic and PJP prophylaxis throughout the treatment period and for 3 months off or until CD4+ count is >200/μL. Antibacterial prophylaxis should be administered for the first month of therapy (when neutropenia is usually unpredictable), in cases of ANC < 500/μL during treatment, in patients with prolonged neutropenia, or at high risk for bacterial infections (for example because of history of recurrent infections), or if prolonged corticosteroid treatment is needed, with discontinuation when risk is reduced, while antifungal prophylaxis is advised in patients with prolonged and severe neutropenia, recent prolonged and/or high dose treatment with corticosteroids, or previous history of fungal infections [9,80,95].

In addition to the above, vaccination strategies play a fundamental role in preventing potentially severe infections [7,8,9,80,95]. At present, it is unclear how much of an impact these therapies have on previously established immunity, but it is known that RRMM patients, particularly after receiving T-cell engaging immunotherapies, often have inferior responses to vaccinations, which should therefore be administered during periods of well-controlled disease, in which a higher immunologic response has been observed) [121,122,123]. In this regard, priority should be given to vaccinations against influenza, SARS-CoV-2, pneumococcal disease and herpes zoster (VZV) [7,95]. Conjugate vaccines, when possible, should be preferred due to higher immunological responses in immunocompromised patients [122,124]. As IgRT may interfere with vaccination efficacy, it is recommended to administer killed or inactivated vaccines >2 months and alive vaccines >8 months after the last supplementation [125,126]. Furthermore, measuring vaccine responses may be useful to assess the need or utility of additional doses, with case-by-case evaluation [122]. As a general rule, vaccinations for endemic or seasonal infections should be started around 3 months after CAR-T infusion; inactivated vaccines and adjuvant vaccines should be administered >6 and >12 months after infusion respectively (the latter only if the patient is no longer considered immunocompromised); live attenuated vaccines can be considered >12–24 months after infusion in absence of residual immunosuppression, due to potential risks [120,122,125]. Patients treated with BsAbs should receive adequate vaccinations with inactivated vaccines (if possible, before the start of therapy), whereas the use of live vaccines is contraindicated [87,95]. Further details regarding specific vaccination recommendations are summarized in Table 4.

Noteworthy, in addition to the aforementioned infection prophylaxis, monitoring, and treatment measures, a modification of the treatment schedule with BsAbs can positively affect reducing infectious risk. Indeed, recent data showed that switching from a weekly to a once-every-two-week administration of BCMA-directed BsAbs can significantly decrease the rate of severe infections, suggesting that a less intensive schedule or fixed-duration treatment might mitigate the infectious risk [127,128]. Notably, in patients receiving BsAbs, the duration of immunosuppression after the end of treatment is currently unknown, highlighting the need to continue adequate infection management even after treatment discontinuation [9].

## 5. Immune Effector Cell Hemophagocytic Lymphohistiocytosis-like Syndrome (IEC-HS)

IEC-HS is a less known, but increasingly recognized toxicity of immune effector cell therapy, recently defined by a dedicated panel of the American Society for Transplantation and Cellular Therapy (ASTCT) as a severe hyperinflammatory syndrome with similar clinical manifestations to hemophagocytic lymphohistiocytosis (HLH) and macrophage activation syndrome (MAS), directly related to CAR-T cell therapy. Therefore, the definition of IEC-HS has substituted the previously described HLH and MAS associated with CAR-Ts. Overall, IEC-HS represents a relatively rare but potentially life-threatening complication of immune effector cell-directed therapies, characterized by signs of macrophage activation and onset or worsening of cytopenias, coagulopathy with fibrinogen consumption, and/or ferritin or transaminase increase [55]. Of note, this toxicity has never been described in association with anti-BCMA or anti-GPRC5D BsAbs [9], although one case related to the anti-CD3/anti-CD19 T cell engager blinatumomab has been reported [129]. By contrast, it has occasionally been described in association with different CAR-T constructs, with experience in MM patients currently limited to clinical trials [1,2,3,25,26,35,37] and few real-life reports [20,130,131].

### 5.1. Incidence of IEC-HS from Clinical Trials and Real-World Setting

The incidence of IEC-HS is relatively low, with 2.9% of patients receiving ide-cel in the KarMMa and KarMMa-3 studies [1,2,132] and 1% of patients receiving cilta-cel in the CARTITUDE-1 and CARTITUDE-4 studies affected [3,25,133]. A retrospective study reported one death due to IEC-HS out of 159 patients who had received ide-cel therapy, which can be roughly considered consistent with the other data available [20]. Data on other CAR-Ts are scarce, but some cases have been reported in phase-one trials with anti-GPRC5D constructs. More specifically, one case of MAS out of seventeen patients receiving MCARH109 [35] and three cases of HLH out of seventy patients receiving BMS-986393 have been described [37]. When examining real-life experiences, a case series on adult patients developing IEC-HS after receiving CAR-T therapy for MM or non-Hodgkin lymphoma reported an incidence of 3% (n = 13/436), including 2 patients with MM [131]. In another case series with more inclusive diagnostic criteria, 12 out of 55 patients (22%) treated with BCMA-CAR-T were diagnosed with MAS, with no defining disease or treatment characteristics that could act as risk factors, although a history of infection prior to CAR-T was more common in affected patients [130].

### 5.2. Pathophysiology and Factors Contributing to IEC-HS

Although the etiopathogenetic process remains to be elucidated, this emergent toxicity is thought to be triggered by tumour antigens activating CAR-T cells, which in turn activate macrophages, leading to a positive feedback loop sustained by cytokine release (including IL-6, IFN-γ, IL-1β, IL-12, IL-18) leading to end-organ damage [55].

Typically, IEC-HS appears after or during CRS, usually 4–10 days after receiving CAR-T therapy, although one case occurring three months after infusion has been reported with cilta-cel [132,133]. Specifically, both case series confirmed that all patients developed CRS prior to IEC-HS and the latter was associated with a longer duration of CRS (5.4 vs. 3.7 days, *p* = 0.03) [130,131]. Moreover, neurotoxicity was also found to be common [130]: in the case of ide-cel, half of the patients (5/10) developing IEC-HS in the KarMMa and KarMMa-3 trials had overlapping neurotoxicity [1,2,132]. Notably, these characteristics seem not to be exclusive of anti-BCMA constructs, as the patient receiving MCARH109 had concomitant CRS and ICANS (both grade 3) [35]. Additionally, patients with IEC-HS had a slower platelet and haemoglobin recovery compared to others, but these differences were resolved three months after CAR-T infusion [130].

### 5.3. Diagnostic Work-Up and Management

IEC-HS diagnosis and prompt identification are made difficult by the absence of specific laboratory alterations or clinical signs, which are often common with CRS or progressive haematological malignancies, and the lack of diagnostic criteria [2,25]. In 2023, however, the ASTCT proposed some consensus criteria for its identification: hyperferritinemia (defined as at least twice the baseline and/or rapidly rising) is required, while the most common manifestations to be considered are cytopenias, decreased fibrinogen, increased transaminases and hemophagocytosis in the bone marrow or other tissues, all with onset when CRS is resolving or has resolved, or after it had initially responded to anti-cytokine therapy. Additional manifestations that can aid in the diagnosis include other coagulation abnormalities, an increase in lactate dehydrogenase, triglycerides or bilirubin, fever, neurotoxicity, kidney failure, or lung manifestations (e.g., hypoxia, oedema, or pulmonary infiltrates). Finally, other causes, most notably CRS, infections, and disease progression, must be ruled out [55].

Likewise, the optimal management for IEC-HS has not yet been precisely defined and refers to local institutional standards [132,133]. Current recommendations include a stepwise approach with treatment aimed at mitigating the cytokine storm and ideally starting as soon as possible to avoid life-threatening complications. In this setting, corticosteroids play a role both in the initial therapy and as a component of subsequent treatments; if not effective, anakinra or ruxolitinib can be considered; if still not effective, other agents such as IFN-γ-targeting empalumab or low-dose etoposide can be considered on a case-by-case basis [7,8,55].

In a real-world experience, all patients had received tocilizumab for prior CRS, and half of them had received systemic steroids to treat CRS, neurotoxicity, or neurotoxicity associated with IEC-HS; ten out of twelve patients received anakinra, with all of them exhibiting a decrease in ferritin levels within three days [11]. The two patients described in the other case series required more aggressive treatment, with both receiving two doses of tocilizumab, prolonged steroid treatment (one patient for 20 days, the other for 50), one dose of siltuximab and supportive care with dialysis; additionally, one required further therapy with cyclophosphamide and ruxolitinib and vasopressor support, and the other received basiliximab [12]. Despite these treatment measures, however, the outcomes were grim, as both patients died by the fiftieth day after CAR-T infusion due to infection or other non-specified causes, regardless of the achievement of complete remission in both cases [131]. Notably, better overall response rates in IEC-HS patients (100% vs. 86%, *p* = 0.05) were also reported in the case series [130]. These data are consistent with those from clinical trials, with one death due to IEC-HS reported in both the KarMMa and CARTITUDE studies. In both studies, the patient who died due to IEC-HS had a concomitant Candida sepsis, or IEC-HS was a contributing factor to the fatal bronchopulmonary aspergillosis [2,25], highlighting the increase in infections, possibly due to the aggressive immune suppressive treatment, also noted from real-world settings [131].

Overall, despite its non-specific clinical manifestations and difficulties in being identified, IEC-HS is gaining increasing recognition, and the uniform and specific definition proposed by the ASTCT is an essential first step towards further research to better define it as a clinical entity and find better treatment approaches.

## 6. Discussion and Future Perspectives

Despite the revolutionary impact of BsAbs and CAR-T cells on MM therapy, their advent has brought new and significant toxicity concerns, necessitating a careful balance between risks and benefits. Given the unprecedented efficacy of these novel T-cell redirecting immunotherapies and the considerable uncertainty surrounding their optimal sequencing, treatment-related toxicities—combined with patient comorbidities and frailty—are playing an increasingly critical role in determining the best therapeutic approach. In addition, the impact of immunotherapy-related toxicities on patient selection will become of increasing magnitude, considering that more and more patients may be potential candidates for such therapies—even in earlier lines—in the near future, and given the increase in treatment options, thanks to the availability of combinations based on the anti-BCMA antibody-drug conjugate belantamab mafodotin, whose toxicity profile appears to be more manageable and better adaptable, even for frail patients [134,135,136,137].

Overall, the data reported so far and discussed in this review delineate a distinct toxicity profile not only between CAR-T and BsAb therapies but also based on the target antigen, albeit a direct head-to-head comparison is lacking. Indeed, CAR-T cells seem susceptible to a higher risk of severe ICAHT, whereas BsAbs appear more burdened by severe infections and persistent hypogammaglobulinemia, especially in patients receiving anti-BCMA-directed therapy. On the other hand, agents targeting the novel antigen GPRC5D—expressed on cells within hard keratinizing tissues and hair follicles—have been associated with new toxicities, including skin and nail changes, rash, and oral-related adverse events such as dysgeusia and dysphagia, reported in studies with both GPRC5D-directed CAR-T cells and BsAbs. As a result, the toxicity profile is a relevant element in the decision-making process and a careful discussion of the potential AEs should be addressed when choosing the treatment plan. As an example, anti-BCMA therapies should be preferred in patients with anamnestic skin disorders or tendencies to be underweight (anti-GPRC5D therapies should be avoided whenever possible in such cases) and prevented in patients with a significant infectious history.

As the prevention and management of AEs during treatment with CAR-T cells and BsAbs in MM patients are becoming increasingly complex, the availability of practical and usable predictive models of severe side effects and complications, as well as clinical outcomes, could represent a useful tool to help directing both therapeutic choice and subsequent management, supporting a risk-adapted approach. In this regard, the CAR-HEMATOTOX score is an important initial step in enabling early risk-stratification of severe toxicity following CAR-T-cell therapy with potential practical applications, while similar predictive models for BsAbs yet are lacking [57]. Nonetheless, distinguishing these side effects from the AEs of chemotherapy or other conventional haematological treatments represents a substantial improvement in the management of these toxicities. Likewise, the standardization of their grading systems allows them to overcome the broad heterogeneity of toxicity reports, enabling data comparison across clinical trials and real-world studies. Indeed, one of the main benefits of the novel EHA/EBMT consensus grading for ICAHT lies in the harmonization of hematotoxicity report with a common nomenclature across different disease contexts and treatment settings, also informing management protocols [12]. In the same way, the 2023 ASTCT consensus diagnostic criteria and grading schema for the new termed IEC-HS can facilitate a prompt diagnosis and provide a uniform approach based on the disease acuity [55].

Furthermore, consensus statements on the prevention and management of these toxicities have recently been released by the EMN and the IMWG Immunotherapy Committee in the past two years to provide guidance and support for the daily management of MM patients under T-cell redirecting agents. However, these recommendations are based on rapidly emerging literature data, not always supported by prospective evidence, as dedicated clinical trials in this setting are lacking. For instance, low-quality evidence supports the preventive role of immunoglobulin replacement therapy; recommended antimicrobial prophylaxis measures were mostly derived from those employed for infection prevention in immunocompromised hosts, while the optimal use of G-CSF to treat severe neutropenia yet is controversial. Therefore, a collaborative approach of data collection through national and international registries as well as the design of future prospective studies to investigate strategies for the prevention and treatment of these AEs, could be helpful in broadening evidence-based knowledge and developing ever-more accurate future guidelines.

Further improved toxicity management is essential, even considering the upcoming advent of novel immunotherapies that simultaneously target multiple pathways, as in the RedirecTT-1 trial, currently exploring a dual-targeting approach in RRMM patients [138]. Additionally, the emergence of trispecific antibodies represents an intriguing and promising frontier in MM treatment [139]. However, while these innovative therapeutic approaches enhance efficacy and overcome resistance mechanisms, they also pose increasingly complex challenges, especially in toxicity management. Meanwhile, significant advances in the management of immunotherapy could arise from the development of new CAR-T constructs, capable of maintaining or even enhancing their anti-tumor efficacy while improving the safety profile of CAR-T-cell therapies. However, larger studies with extended follow-up are needed to fully determine the true potential of these promising new products in reducing not only the incidence and severity of CRS and ICANS but also other long-term treatment-related toxicities [10,11,140,141].

In the context of ICAHT, key areas for further investigation include the identification of early causes of ICAHT, understanding the causal role of pro-inflammatory biomarkers, such as IFN-γ and IL-6, and of clonal hematopoiesis in the development of cytopenias, and exploring the relationship between ICAHT and treatment-emergent myeloid neoplasms. Furthermore, alternative lymphodepletion regimens that could mitigate cytopenias should be explored, and the optimal sequencing and timing for G-CSF, TPO-RA, and autologous or allogeneic hematopoietic cell boosts should be determined [43].

Infections remain one of the major concerns in patients treated with CAR-T cell therapies, particularly in those receiving anti-BCMA bispecific antibodies, due to the off-target B-lymphocyte depletion effects that persist throughout treatment. Overall, bacterial and viral infections are the most common, whereas fungal infections are relatively rare, with respiratory tract infections representing the main type of infectious complications. However, detailed information regarding the infectious risk and the specific pathogens involved remains limited, as such data are inconsistently reported across clinical trials and real-world studies, underscoring the urgent need to enhance the clarity and accuracy of infection reporting. In this regard, the recent proposal by Ludwig et al. for a standardized model to report infections in RRMM patients treated with BsAbs represents an initial valuable effort [100]. Similarly, the actual impact of currently recommended prophylactic strategies remains to be defined, particularly considering the growing emergence of epidemiologically variable antibiotic resistance. In line with this, the efficacy of vaccination in the novel immunotherapy era, along with the optimal timing for administration and the impact of booster doses, remains largely unexplored. Moreover, given the wide range of patients for whom these therapies are intended, and the logistical and economic commitment required, the role of immunoglobulin supplementation to mitigate the infectious risk during prolonged hypogammaglobinemia should be confirmed prospectively, as well as the best route and frequency of administration. Notably, there is significant interest in determining whether fixed duration or response-directed duration approaches in patients receiving BsAbs could limit the development of toxicities.

While improvements are anticipated with increased awareness, vigilance, and the implementation of new expert management guidelines, strict infection surveillance remains essential throughout the entire course of treatment. In cases of common clinical symptoms such as suspected pneumonia or urinary tract infections, appropriate empirical antibiotic therapy should be promptly initiated, together with standard diagnostic procedures, including blood, urine, and sputum cultures, as well as molecular panels for respiratory viruses. If no pathogen is identified and clinical improvement is lacking, further evaluation for atypical and opportunistic infections is warranted. Notably, the effective management of suspected infections in patients undergoing T-cell redirecting therapies relies on close collaboration between haematologists and infectious disease specialists to ensure timely and targeted interventions.

Concerning IEC-HS, although a major step has been taken in recognizing it as a specific clinical entity, several knowledge gaps need to be filled at levels of etiopathogenic processes, inflammatory patterns, risk factors and treatment approaches to improve patient outcomes.

## 7. Conclusions

The advent of new immunotherapies has certainly disrupted the therapeutic landscape for patients with RRMM with unprecedented results. In parallel, the unique toxicities that have emerged with these agents have posed equally impactful challenges, requiring major efforts to prevent and minimize the related AEs and optimize patient care. Indeed, with the rapid ascent of the learning curve on the management of the now well-known CRS and ICANS, other frequently encountered specific toxicities such as ICATH, hypogammaglobinemia, infections, as well as the less frequent IEC-HS, initially underestimated, have emerged as less and less non-prototypical events of T-cell redirection therapies, resulting in high long-term resource expenditure and potentially life-threatening morbidity and mortality consequences.

Nowadays, optimal management of such side effects becomes mandated so as not to compromise the efficacy benefits achieved so far, patient quality of life, and prospects for further treatment. In addition, improvements in toxicity management could help to reduce disparities in access to MM treatment, with the widespread use of both classes of therapies in an ever-widening audience of patients who could receive these therapies safely and effectively in the outpatient setting, instead of being restricted to selected referral centres, and ensure more informed use of new combinations, or new agents targeting multiple pathways simultaneously.

## Figures and Tables

**Table 1:** Grading of early and late ICAHT according to EHA/EBMT consensus [12].

Grading	1	2	3	4
Early ICAHT				
ANC ≤ 500/μL	≤7 days	7–13 days	≥14 days	Never > 500/μL
ANC ≤ 100/μL	/	/	7–13 days	≥14 days
Late ICAHT				
ANC	≤1500/μL	≤1000/μL	≤500/μL	≤100/μL

Abbreviations: ANC = absolute neutrophil count.

**Table 3:** Summary of recommendations for cytopenias and hypogammaglobulinemia (references in text).

CAR-T-Cell Recipients	BsAbs Recipients
**Anaemia**	
Transfusion of packed RBC concentrates as per institutional standards Erythropoiesis stimulating agents if long lasting/severe anemia as per institutional guidelines	Transfusion of packed RBC concentrates if G ≥ 3 or symptomatic patient Erythropoiesis stimulating agents
**Thrombocytopenia**	
Transfusion of PLT concentrates as per institutional standards Thrombopoiesis stimulating agents if long lasting/severe thrombocytopenia as per institutional guidelines	Transfusion of PLT concentrates if G4 without bleeding or G3 with bleeding Hold BsAb administration until PLT > 50,000
**Neutropenia**	
Prophylactic G-CSF from day +2 if high risk of severe ICAHT (high HT score) Therapeutic G-CSF if ANC < 500/μL If G-CSF refractory: autologous or allogeneic HCB and TPO-RA If no response: allo-HCT	G-CSF if G ≥ 3 neutropenia (to be avoided when pts are at high risk for CRS) If G4 or febrile neutropenia: BsAb discontinuation until resolution
**Hypogammaglobulinemia**	
IgRT (400–500 mg/kg every 4 weeks) if serum IgG < 400 mg/dL or if severe and/or recurrent infections	

Abbreviations: CAR-T = chimeric antigen receptor T-cells; BsAbs = bispecific antibodies; RBC = red blood cell; G = grade; PLT = platelet; G-CSF = granulocyte colony-stimulating factor; ICAHT = immune effector cell associated hematotoxicity; HT = CAR-HEMATOTOX; ANC = absolute neutrophil count; HCB = haematopoietic cell boost; TPO-RA = thromopoietin receptor agonist; allo-HCT = allogeneic haematopoietic stem cell transplantation; CRS = cytokines release syndrome.

**Table 4:** Summary of recommendations for antimicrobial prophylaxis (references in text).

CAR-T-Cell Recipients	BsAbs Recipients
**Herpes prophylaxis**	
Aciclovir or valaciclovir: all pts from LD until 1 yr after infusion and/or CD4+ count > 200/μL	Aciclovir or valaciclovir: all pts until 3 mos off-therapy and CD4+ count > 200/μL
**PJP prophylaxis**	
Co-trimoxazole: all pts from LD until 1 yr after infusion and/or CD4+ count > 200/μL	Co-trimoxazole: all pts throughout treatment period until CD4+ count > 200/μL
**Antibacterial prophylaxis**	
Specific drug based upon local bacterial epidemiology: high risk of severe ICAHT and ANC < 500/μL	Specific drug based upon local bacterial epidemiology: first months of therapy; ANC < 500/μL; prolonged neutropenia; high risk of bacterial infections
**Antifungal prophylaxis**	
Specific drug based upon individual risk: high risk of severe ICAHT and ANC < 500/μL; high risk of fungal infections (concomitant steroids, prior allo-HCT, prior invasive aspergillosis)	Specific drug based upon individual risk: high risk of fungal infections (prolonged and severe neutropenia, prolonged and/or high dose corticosteroids, prior fungal infection)
**CMV prophylaxis**	
Not recommended	Not recommended
**HBV prophylaxis**	
Entecavir or tenofovir: HBV carriers or previous HBV infection	Entecavir or tenofovir: HBV carriers or previous HBV infection
**Recommended vaccinations**	
- Influenza: ≥2 weeks before LD, booster 3–6 mos after infusion, then annually - SARS-CoV-2: ≥2 weeks before LD, then full revaccination starting 3 mos after infusion - VZV recombinant adjuvanted 2–12 mos after infusion (particularly if seropositive, history of chickenpox or shingles) - HBV > 6 mos after infusion if lacking seroprotection - PCV20: > 6 mos after infusion - DTap > 6 mos after infusion, with 2 DT further doses over 6–12 mos - Hib and meningococcal if additional risk factors (e.g., functional asplenia)	- Influenza (yearly prior to onset of winter) - SARS-CoV-2 - PCV20 - VZV recombinant adjuvanted (particularly if seropositive, history of chickenpox or shingles) - Hib and meningococcal if additional risk factors (e.g., functional asplenia and hypogammaglobulinemia)

Abbreviations: CAR-T = chimeric antigen receptor T-cells; BsAbs = bispecific antibodies; pts = patients; LD = lymphodepletive chemotherapy; yr = year; mos = months; PJP = pneumocystis jirovecii; ICAHT = immune effector cell associated hematotoxicity; ANC = absolute neutrophil count; allo-HCT = allogeneic haematopoietic stem cell transplantation; CMV = cytomegalovirus; HBV = hepatitis B virus; VZV = varicella zoster virus; PCV20 = 20-valent pneumococcal conjugate; DTap = diphtheria/tetanus/acellular pertussis; Hib = Haemophilus influenza type b.
